# Influence of a 2-week transcutaneous auricular vagus nerve stimulation on memory: findings from a randomized placebo controlled trial in non-clinical adults

**DOI:** 10.1007/s10286-024-01053-0

**Published:** 2024-07-22

**Authors:** Veronika Cibulcova, Julian Koenig, Marta Jackowska, Vera Kr Jandackova

**Affiliations:** 1 Department of Epidemiology and Public Health, Faculty of Medicine, University of Ostrava, Syllabova 19, Ostrava, 703 00 Czech Republic; 2 Department of Child and Adolescent Psychiatry, Psychosomatics and Psychotherapy, Faculty of Medicine, University of Cologne, University Hospital Cologne, Cologne, Germany; 3 Faculty of Psychology, SWPS University, Sopot, Poland; 4 Department of Human Movement Studies, Faculty of Education, University of Ostrava, Ostrava, Czech Republic

**Keywords:** Immediate recall, Memory, Memory modulation, Transcutaneous vagus nerve stimulation, Vagus nerve

## Abstract

**Purpose:**

Memory plays an essential role in daily life and is one of the first functions to deteriorate in cognitive impairment and dementia. Transcutaneous vagus nerve stimulation (tVNS) is a promising therapeutic method; however, its ability to enhance memory is underexplored, especially considering long-term stimulation. We aimed to investigate the effect of a 2-week course of auricular tVNS (taVNS) on memory in a non-clinical population.

**Methods:**

This single-blind randomized placebo-wait-list controlled trial recruited 76 participants (30 men; mean age 48.32 years) and randomized them into four groups: early active/sham taVNS and late active/sham taVNS. Participation in the study lasted 4 weeks; early groups underwent 2 weeks intervention immediately following the first study site visit (days 0–13) and late groups 2 weeks after the first study site visit (days 14–27). Active and sham taVNS included 2 weeks of daily 4-h neurostimulation at the tragus or earlobe, respectively. To assess memory, we used the Rey Auditory Verbal Learning Test.

**Results:**

Two weeks of active taVNS, but not sham taVNS, improved immediate recall and short-term memory score both in early and late groups. Furthermore, the improvements persisted over subsequent follow-up in early active taVNS. Importantly, the effect of active taVNS was superior to sham for immediate recall in both early and late groups. There were no statistical differences in delayed recall.

**Conclusion:**

Our findings suggest that taVNS has potential to improve memory, particularly immediate recall, and may be an effective method in preventing memory loss and mitigating cognitive aging.

**Supplementary Information:**

The online version contains supplementary material available at 10.1007/s10286-024-01053-0.

## Introduction

Diseases associated with memory impairment, particularly Alzheimer’s disease and other neurodegenerative diseases, are of increasing clinical importance due to the current ageing population [1]. There is a growing interest in the relationship between autonomic nervous system (ANS) dysregulation and cognitive function [2]. Allan et al. showed autonomic dysfunction in the most common dementia subtypes (Alzheimer’s disease, vascular dementia, dementia with Lewy bodies, and Parkinson’s disease dementia) compared to healthy controls of the elderly [3]. Changes in sympathetic/parasympathetic balance are thought to play a role in cognitive impairment, especially in executive function, learning, and memory tasks [2]. Recent systematic reviews and meta-analyses of 18 studies found moderate associations between higher heart rate variability (HRV), a non-invasive biomarker of ANS function, and better cognitive and behavioral outcomes in neurodegenerative disorders and dementia [4, 5]. Both vagally mediated HRV indices, namely the root mean square of successive differences between normal heartbeats (RMSSD) and high frequency power (HF), were associated with cognitive/behavioral outcomes. Similarly, a meta-analysis of 24 studies [4] showed the same vagally mediated HRV measures, HF and RMSSD, to be significantly lower in the dementia group than in the healthy group. Results of studies in healthy adults also revealed higher HRV to be associated with better cognitive function [6]. Further, reduced cognitive performance was observed in participants with lower HRV [7].

The vagus nerve is an important component of the parasympathetic nervous system. Given the widespread projections of the vagus nerve and its involvement in ANS function, it is not surprising that vagus nerve stimulation (VNS) has been proposed to have great potential for treating ANS-related disorders [8]. Afferent vagal nerve fibers have extensive projections from the nucleus solitary tract (NST) to the locus coeruleus (LC), the brain’s primary noradrenergic center, which in turn projects to key brain regions such as the amygdala, hippocampus, thalamus, hypothalamus, basal forebrain, and prefrontal cortex. These areas are crucial for memory, sensory processing, wakefulness, stress response, alertness, arousal, attention, and higher-order cognitive functions, thereby allowing the vagus nerve to modulate various behavioral processes involved in learning and memory [9]. Importantly, vagal afferent fibers are hypothesized to be responsible for the hippocampus volume effect in older individuals when heart rate oscillation biofeedback is used [10]. This explains why several studies have highlighted the effects of VNS on memory performance. VNS can be delivered invasively (iVNS) or non-invasively, with auricular tVNS (taVNS), and cervical tVNS (tcVNS), which is known to stimulate both efferent and afferent fibers [11]. Several studies have shown promising effects of chronic iVNS in clinical populations, for instance in patients with Alzheimer’s disease [12, 13] or depression [14]. Patients treated with iVNS for treatment-resistant depression have shown improvements in learning and memory after 1 month, and these cognitive benefits have been maintained for up to 2 years [14]. However, iVNS involves surgical implantation of a pulse generator in the left thoracic region [15], and can lead to surgical complications and unwanted side effects [16]. tVNS involves stimulating vagus afferent fibers through electrodes placed on the skin in areas where the vagus nerve is distributed. Research findings suggest that tVNS yields similar effectiveness as iVNS, but with reduced side effects and enhanced tolerability [17, 18]; for example, a study by Murphy et al. showed promising effects of tVNS on brain connectivity in patients with mild cognitive impairment [19].

Several studies [11, 20–29] have explored the effects of tVNS on cognitive function in healthy volunteers, aiming to replicate the cognitive effects observed in patients who underwent iVNS. Meta-analysis of short-term tVNS in young healthy adults has found an overall moderate effect for improved cognitive performance, particularly executive function [30]. However, comprehensive long-term studies in non-clinical populations are still missing, limiting the generalization of tVNS effects to non-clinical adults and its role in prevention of memory problems and potential benefits in slowing down the process of memory ageing. Therefore, given the growing interest in non-invasive and portable devices that stimulate the vagus nerve [31], and the need to test novel techniques that could improve cognitive function in non-clinical adults, we conducted a randomized placebo wait-list controlled trial in non-clinical adults aged 18–75 years. The current study investigated the effect of a non-invasive, 2-week course of taVNS on verbal memory. We hypothesized that taVNS would improve memory performance.

## Methods

### Design

We used data from a single-blinded, randomized, placebo wait-list controlled trial (NCT04070547, registered at https://clinicaltrials.gov) that assessed the effect of a 2-week course of taVNS on cognitive function and health-related variables. Participation in the study lasted 4 weeks, including 2 weeks of intervention using a stimulation device. The focus of the present manuscript is on memory, which was assessed on each testing day (three study site visits). The trial is reported in accordance with the Consolidated Standards of Reporting Trials (CONSORT2010 Statement) [32].

### Participants

Seventy-eight participants (31 men, 47 women), aged 18–75 years, were recruited through announcements (e.g., via e-mail) and flyers at the University of Ostrava (Czech Republic) and neighboring institutions from 2018 to 2019. As a result of the intricate study design, the sample size was not determined through a formal a priori power analysis but in line with existing research, e.g., Bretherton et al. [33] and Jacobs et al. [25]. Participants were screened by phone, with the following inclusion criteria: (1) apparently healthy status, (2) age between 18 and 75 years, and (3) speaking Czech. Exclusion criteria were (1) history of cardiovascular disease, (2) major mental disorders, (3) severe inflammation, (4) severe neurological disorders, (5) taking medication that may affect autonomic pathways, (6) brain surgery, or (7) pregnancy (for more details visit https://clinicaltrials.gov). Written informed consent was obtained from all participants prior to participation in accordance with the ethical principles of the Declaration of Helsinki. The protocol was approved by the Ethics Committee of the University of Ostrava. Participants who completed the study received information on their individual test scores and a honorarium of CZK 1000.

### Randomization, masking and allocation concealment

Participants underwent a two-stage randomization process. Participant allocation was undisclosed until their inclusion in the study was confirmed. Simple randomization, using a shuffled deck of cards, determined group assignments. In the first stage, before their initial lab visit, they were randomly assigned to either the early or late phase (with a 1:1 allocation ratio) by the project’s lead investigator, who was minimally involved in data collection. After the participant signed the informed consent, the laboratory staff received a check-list (with standardized exact instructions for the measurement process and procedure and an ID identifier) with information about the allocation (early or late). The second randomization occurred at the end of the pre-intervention session after participants completed all assessments and it was time to deliver the stimulator to the participant. This step divided participants into active or sham taVNS groups (with a 1:1 allocation ratio). This assignment was facilitated by laboratory staff who drew a card from a deck within a non-transparent cabinet, with a red card indicating active taVNS and a black card signifying sham taVNS group. Early phase (active or sham) participants started the intervention immediately after the first visit (days 0–13), and late (wait-list) phase participants began 2 weeks later (days 14–27). The randomization into the early and late phases allowed us to evaluate changes in memory performance. This included comparisons between the pre- and post-intervention phases in both the active and sham groups. Additionally, we aimed to assess changes in memory performance after the cessation of active or sham taVNS in the early phase (days 14–27), as well as changes during the waiting period (without any intervention) in the late phase (days 0–13).

Participants were blinded to their stimulation group (active or sham). To conceal the allocation from participants, the name and identifiers of the taVNS stimulator were masked, making it difficult for participants to find information about the stimulator on the internet. They were informed that they belonged to one of several groups, which differed in the placement of electrodes on the ear, as we were evaluating the effectiveness of various ear sites. Double-blinding was not conducted in our study because the laboratory staff, extensively trained in science ethics and standardized memory testing, were four researchers who also had thorough knowledge of taVNS and were familiar with the locations used for active and placebo stimulations from previous studies. Laboratory staff provided instructions to participants on how to properly fit and use the stimulator and gave the participants a manual with these instructions. After the intervention period, laboratory staff verified the correct use of the stimulator by asking participants to demonstrate its application and settings. Statistical analyses were conducted independently by two members of our team who were either not involved at all or minimally involved in data collection.

### Procedure

**Fig. 1:**
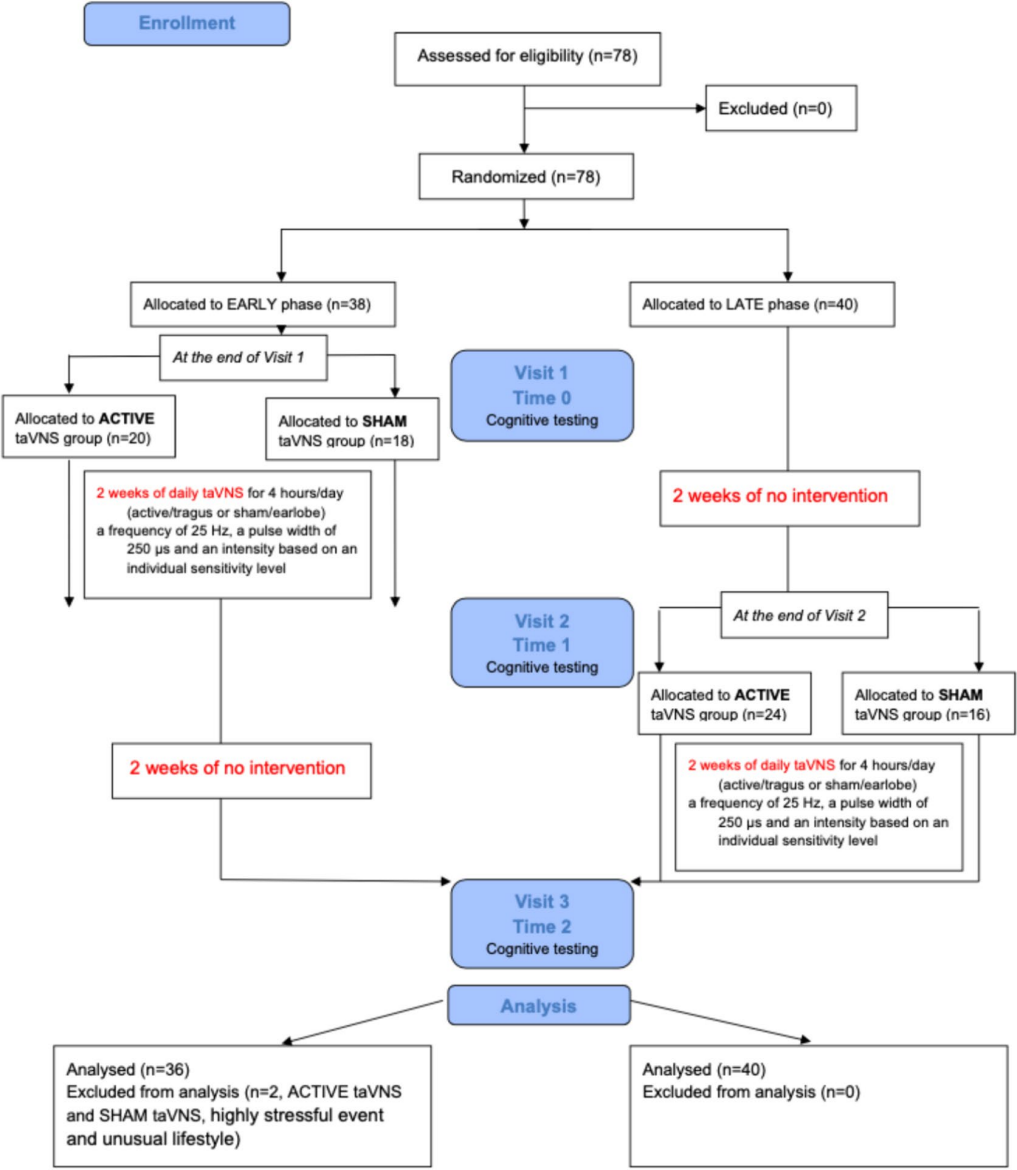
CONSORT flowchart

Participants completed sociodemographic and other questionnaires regarding health and prescribed medications via the Qualtrics platform before their first visit. They received an explanation of the procedure and possible adverse effects at the beginning of the first experimental session. No information was provided about the different types of stimulation (active or sham) and hypothesized outcome effects. Before each visit, participants were asked to adhere to certain conditions, such as following a normal sleep schedule the day before the experiment, eating a light breakfast or lunch on the day of the laboratory session, avoiding alcohol and strenuous exercise 24 h before the visit, and avoiding coffee or caffeinated beverages such as energy drinks and smoking at least 2 h before the visit. At the beginning of each session, it was further ascertained if participants met all inclusion criteria so as to exclude potentially confounding variables. Body weight and waist-hip circumference were measured once. A graphical overview of the experimental procedure is shown in Fig. 1.

All the experimental sessions were conducted at the Psychophysiology Laboratory at the University of Ostrava in a quiet examination room by a trained laboratory staff member. Participants were invited three times at a similar time of the day (if it was possible), separated by 2 weeks (± 1 day). The measurement took place between 10:00 and 17:00, depending on the availability of the participant.

### Measures

#### Sociodemographic and health-related characteristics

Age, sex, education level (high-college or university vs. lower education), and employment status were measures of sociodemographic and economic information (Table 2). Height and weight were collected to calculate BMI (kg/m^2^). We also collected self-reported information on whether participants were taking any prescribed medication. We focused on the usage of antihypertensives, diabetes medication, antidepressants, anxiolytics, and hypnotics, which are thought to interact with autonomic pathways (see statistical analysis and Table 2). Depressive symptoms were measured using a 10-item version of the Center for Epidemiological Research Depression Scale (CES-D) [34]. A more thorough explanation of the use of CES-D in this study may be found in another research paper [35]. Prevalent health issue was defined when the participant self-reported “yes” to the question “Do you have any chronic medical limitations diagnosed by a doctor”?

#### Memory assessment

We used the Czech version [36] of the Rey Auditory Verbal Learning Test (RAVLT) to measure verbal learning and memory. We explained the procedure in detail to the participants before the test to reduce the first session disadvantage. Briefly, the RAVLT consists of presenting the participant with a list of 15 semantically unrelated words read aloud with clear articulation at a rate of one word every 2 s. The participant is instructed to remember as many words as possible and to repeat them immediately after the words are read regardless of the order in which they are presented. This procedure is repeated five times with a list of words from set A (RAVLT I, RAVLT II, RAVLT III, RAVLT IV, RAVLT V). Instructions are repeated before each trial. An index of immediate recall is provided by the score RAVLT I. The five recall trials (RAVLT I–V) are summed into one short-term memory score. The examiner then presents a second list of 15 words (set B, interference list), giving the participant only one attempt to remember and recall this new list (RAVLT VI). Immediately afterward, the participant is asked to recall as many words as possible from the first list (RAVLT VII). After approximately 30 min (filled in by computerized tests of executive function), the participant is again asked to recall as many words as possible from the first list (RAVLT VIII, delayed recall). Taking into account the practice effect, one original and two alternative versions of the Neuropsychological Battery of the Prague Psychiatric Centre [37] were used. All participants were assessed with all three versions of the test (A–C) in a random order throughout the three laboratory sessions (a shuffled deck of playing cards was used).

Our main outcomes in subsequent analyses were immediate recall (RAVLT I), short-term memory score (RAVLT I–IV), and delayed recall (RAVLT VIII).

### Auricular transcutaneous vagus nerve stimulation

taVNS was delivered using a Parasym® PK01 neurostimulation device (Parasym Ltd, London, UK). The taVNS stimulation device consists of a stimulation unit that generates an electrical current and two gold-plated metal ear electrodes that transmit electrical impulses from the stimulator to the skin surface on the inner and outer surfaces of the tragus or earlobe of the ear. The device is CE marked.

**Fig. 2:**
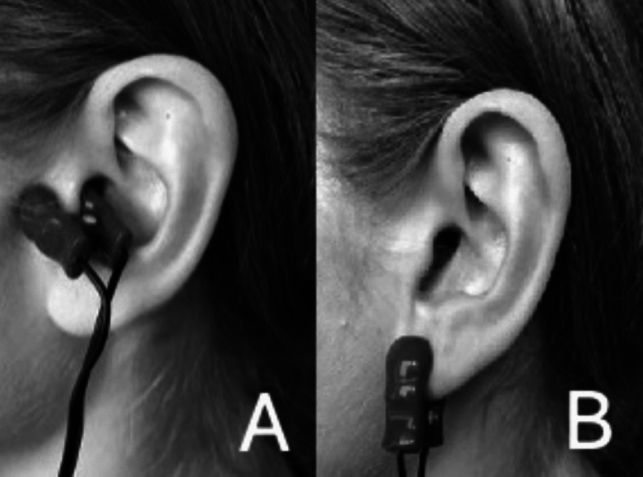
**A** active taVNS (tragus), **B** sham taVNS (earlobe)

For the experimental condition (active taVNS, *N* = 43), the stimulation electrodes were applied to the inner and outer surface of the left tragus (Fig. 2A). This was based on knowledge of the distribution of the auricular branch of the vagus nerve (ABVN) [38], and on evidence from a functional magnetic resonance imaging (fMRI) study that confirmed activation of cortical regions in the vagal afferent pathway after tragus stimulation compared with sham stimulation [39]. As a result of concerns about cardiac safety [40], we used only the left side for taVNS, which was standard practice at the time of planning and conducting our study. This practice was also required by the manufacturer in the user manual [41]. Furthermore, only left-sided stimulation has been approved by the US Food and Drug Administration (FDA) for iVNS [42]. However, recent scientific discussions have questioned whether these conventional reservations about taVNS are justified [43, 44].

In the sham (placebo) condition (*N* = 33), stimulation electrodes were placed on the center of the left earlobe (Fig. 2B). Earlobe stimulation produces the same tingling sensation as active stimulation, although the earlobe has been found to be free of cutaneous vagal innervation [38].

Participants were asked to use the device for 4 h (240 min) per day (only during daytime), regardless of the number of sessions or time of day (however, it was recommended not to stimulate for 4 h continuously because of the higher risk of contact pain). Laboratory staff instructed the participants to use the device with a CONST mode (constant stimulation), a frequency of 25 Hz, and a pulse width of 250 μs. We employed a pulse width of 250 μs and a frequency of 25 Hz, parameters widely utilized in studies investigating the effects of taVNS on cognition [45]. While 25 Hz stimulation specifically has been shown to activate the LC [46] and NST [47], frequencies between 20 and 30 Hz have been approved by the FDA for clinical use [48] and indeed over 100 neuroimaging and taVNS trials indicate that this commonly used frequency range demonstrates biological activity [48, 49].

While the mode, frequency, and pulse width settings remain stored in the stimulator, the stimulation intensity must always be reset. Participants were instructed to individually set the taVNS stimulation intensity to their sensory threshold using a staircase procedure (increasing it by one level per second as recommended by the manufacturer [41]) until they felt a “tingling” sensation. The stimulus was then adjusted to be barely perceptible and comfortable (clearly below their pain threshold). Participants were informed that they could readjust the intensity during the stimulation session if they no longer felt the tingling. Using this method should lead to the recruitment of the thickly myelinated Aβ fibers, responsible for mechanoreception and touch sensation, rather than Aδ fibers, which sense pain and temperature. To achieve this effect, the stimulus must be delivered at lower current intensities, always below the pain threshold [50] because the small diameter Aδ and C fibers require significantly higher stimulus intensities for depolarization than the thickly myelinated, larger diameter Aα or Aβ fibers [51]. By this approach we also minimized pain and ensure participant comfort. The intensity of the Parasym device ranges from 1 to 36 mA, and the waveform was the Parasym proprietary waveform. Participants were asked to clean the site and the electrodes and use a conductive spray before each stimulation session. The initial device setting was done with the help of a laboratory staff member at the end of a pre-intervention session.

The aforementioned taVNS device settings are reported according to the international consensus for minimum reporting standards [52] and are summarized in Table 1.

**Table 1:** Stimulation parameters in accordance with minimum reporting standards [52]. Adapted from [53]

Stimulation parameter	Details
Device manufacturer	Parasym Ltd, London, UK, model Parasym PK01
Regulatory aspects	CE certification
Stimulation site	Left tragus (active taVNS) or left earlobe (sham taVNS); anterior and posterior surface
Intensity level	Individual, based on the perception threshold, maximum 36 mA (across 500 Ω load, RMS value 6.08 mA)^a^
Electrodes	Two gold-plated metal, ear-clipped
Duty cycle	Constant stimulation, with no ON/OFF cycling
Frequency	25 Hz (1–30 Hz adjustable)
Pulse shape	Rectangular
Pulse width	250 μs (50–250 μs adjustable)
Waveform	Parasym proprietary waveform^a^
Stimulation period	4 h/day, ~ 14 days

^a^Information provided by manufacturer

#### Adherence

We measured participant adherence through online daily questionnaires, where they reported daily taVNS stimulator usage details. Additionally, at post-intervention visit, participants anonymously indicated the days and daily duration of taVNS use on colored paper (yellow for sham, white for active), deposited in a cardboard box. After the 2-week taVNS course, the participants were also asked to demonstrate how they used the stimulator in order to determine whether it was used correctly.

### Statistical analyses

Seventy-eight participants were initially enrolled, but two were excluded—one because of a highly stressful event and another with an unusual lifestyle. Therefore, 76 participants were included in the analyses. Participant characteristics are presented as frequencies, means, and 95% confidence intervals. Group differences were assessed using chi-squared test (categorical variables), analysis of variance (ANOVA; continuous variables, normally distributed), or Kruskal–Wallis tests (continuous variables, non-normally distributed). Shapiro–Wilk test was used to test the normality of the distribution. Spearman rank correlation coefficients (*r*_s_) examined memory outcome correlations.

In order to test the simple effect of taVNS on memory outcomes we performed mixed effect regression modelling and contrast analysis. Mixed effect models are robust tests that use all available data over the follow-up period and consider the fact that repeated measures on the same individual are dependent. In these analyses, the intercept was fitted as a random effect, based on participant IDs. This approach allows each participant to have their own baseline level of the memory outcome variable. The three memory outcomes were examined in separate models. An intraclass correlation coefficient (ICC) of 0.6, 0.8, and 0.7 indicated that the mixed model was useful and necessary. The basic model for each memory outcome included the following terms: memory variable of interest (immediate recall, short-term memory score, or delayed recall), time (T0—visit 1, T1—visit 2, T2—visit 3), phase (early or late) and intervention (active or sham taVNS) and two-way interaction terms time × intervention, time × phase, and intervention × phase; along with the three-way interaction time × intervention × phase to explore the nuanced effects of these variables and their interplay. Each model was adjusted for sex (categorized as men or women) and age (treated as a continuous variable) as covariates to control for potential confounding effects. In our analyses, contrasts were first derived a priori from the respective mixed linear models, in order to investigate changes between adjacent timepoints (mainly T0 vs. T1 in early phases, T1 vs. T2 in late phases) for each interaction of intervention type (active or sham taVNS) and phase (early or late). The use of contrasts has been suggested as an effective way to test a priori expectations from complex statistical models such as ours where a three-way interaction term is used. Planned comparisons between specific conditions or clusters of conditions are recommended to be implemented as contrasts [54, 55].

In the main analysis, in the case of statistical differences for one intervention condition (active or sham taVNS) between adjacent time periods per expectation in the group (i.e., when actual stimulation was applied), superiority of the effect was tested against the other intervention condition (sham or active taVNS). To accomplish this, a statistical approach based on Wald test principles (often referred to as chi-square statistics in this context) was adopted.

To visualize differences between pre- and post-intervention values, we calculated changes in selected memory scores at time 1 compared to time 0 for the early phases and at time 2 compared to time 1 for the late phases. Analysis of covariance, controlled for age and sex, with pairwise comparisons from contrast analysis compared changes between active and sham taVNS for early and late phases separately.

Additional models included self-reported presence of health issue and prescribed medication use including hypnotics, anxiolytics, and antidepressants. Sensitivity analysis excluded participants using these medications. For all analyses STATA/IC 16.1 for Mac (StataCorp LLC, College Station, Texas, USA) was used, with a significance level set at *p* < 0.05.)

## Results

### Description of the sample

Table 2 presents baseline characteristics of the participants in each of the four groups. Only very small differences were observed between the groups. In the statistical evaluation, no differences in sociodemographic, health-related variables, or baseline memory scores were found between the four groups (*p* > 0.157) and between the early and late phases (*p* > 0.125). In the entire analytical sample, the correlation between the three memory scores was moderate to strong: immediate recall vs. short-term memory score was *r*_s_ = 0.78, short-term memory score vs. delayed recall was *r*_s_ = 0.79, and immediate recall vs. delayed recall was *r*_s_ = 0.52 (*p* < 0.0000 for all correlations).

**Table 2:** Baseline characteristics of 76 participants in the four experimental conditions

	Early		Late	
	Active taVNS	Sham taVNS	Active taVNS	Sham taVNS
Sample size (*N*)	19	17	24	16
Age	45.1 (35.5–54.6)	52.8 (43.4–62.1)	50.5 (43.9–57.2)	44.1 (33.8–54.4)
Sex				
Male	9 (47.37)	7 (41.18)	8 (33.33)	6 (37.50)
Female	10 (52.63)	10 (58.82)	16 (66.67)	10 (62.50)
High educational degree	6 (33.33)	11 (64.71)	14 (58.33)	10 (62.50)
Employment status				
Student	6 (33.33)	2 (16.67)	2 (10.00)	4 (28.57)
Employed	7 (38.89)	8 (66.67)	17 (85.00)	8 (57.14)
Others (retired, self-employed)	5 (27.78)	2 (16.67)	1 (5.00)	2 (14.29)
Body mass index	25.0 (22.5–27.5)	26.7 (24.4–29.0)	25.8 (24.0–27.6)	26.1 (23.8–28.4)
Prescribed medication use	6 (31.58)	7 (41.18)	10 (41.67)	4 (25.00)
Antihypertensives	2 (10.53)	5 (29.41)	5 (20.83)	1 (6.25)
Diabetes medication	1 (5.26)	1 (5.88)	1 (4.17)	0 (0)
Antidepressant use	0 (0)	1 (5.88)	1 (4.17)	1 (6.25)
Anxiolytics	0 (0)	0 (0)	1 (4.17)	0 (0)
Hypnotics	0 (0)	0 (0)	1 (4.17)	0 (0)
CES-D^a^	7.39 (5.1–9.7)	4.94 (3.58–6.30)	6.68 (5.03–8.34)	7.25 (4.73–9.77)
Self-reported diagnosed health issue	6 (33.33)	3 (17.65)	6 (25.00)	1 (6.25)
Main outcomes				
Immediate recall ^b^	7.74 (6.49–8.98)	7.76 (6.39–9.14)	7.42 (6.66–8.17)	8.38 (7.33–9.42)
Short-term memory score^b^	57.21 (53.08–61.34)	55.76 (50.75–60.78)	54.88 (51.00–58.75)	59.31 (55.16–63.46)
Delayed recall^b^	12.11 (10.94–13.27)	11.06 (9.43–12.69)	11.5 (10.35–12.65)	13 (11.62–14.38)

Data are presented as* n* (%) or mean (95% CI), unless otherwise indicated

^a^Derived from 10-item version of the Center for Epidemiological Research Depression Scale

^b^Derived from Rey Auditory Verbal Learning Test

#### Adherence

According to the anonymous box data, participants reported using the taVNS device for at least 12 of the 14 days of the intervention. For the active taVNS group, the average daily use was 3.8 h, compared to 3.9 h for the sham taVNS group. Participants in the active taVNS group anonymously reported 95% daily use of the taVNS device, compared to 92% in the sham taVNS group. Additionally, Table 3 presents results on adherence based on daily online questionnaires.

**Table 3:** Adherence data from daily online questionnaires

	Early		Late		*p* value
	Active taVNS	Sham taVNS	Active taVNS	Sham taVNS	
Hours of stimulation/day	3.58 (3.25–3.91)	3.83 (3.69–3.97)	3.47 (3.01–3.92)	3.49 (3.25–3.74)	0.285
Days	13.84 (13.51–14.17)	13.88 (13.28–14.48)	13.08 (12.02–14.15)	13.63 (12.90–14.35)	0.826
Stimulation periods	3.74 (3.29–4.20)	3.96 (3.65–4.27)	4.09 (3.77–4.40)	4.03 (3.62–4.44)	0.819

Data are presented as mean (95% CI)

### Within-individual changes in memory outcomes

On average, results from the mixed models indicated a main effect of time on immediate recall (*p*_Wald test_ = 0.023) and short-term memory score (*p*_Wald test_ = 0.001); both measures increased over the three laboratory sessions. There was no main effect of time on delayed recall (*p*_Wald test_ = 0.785).

**Fig. 3:**
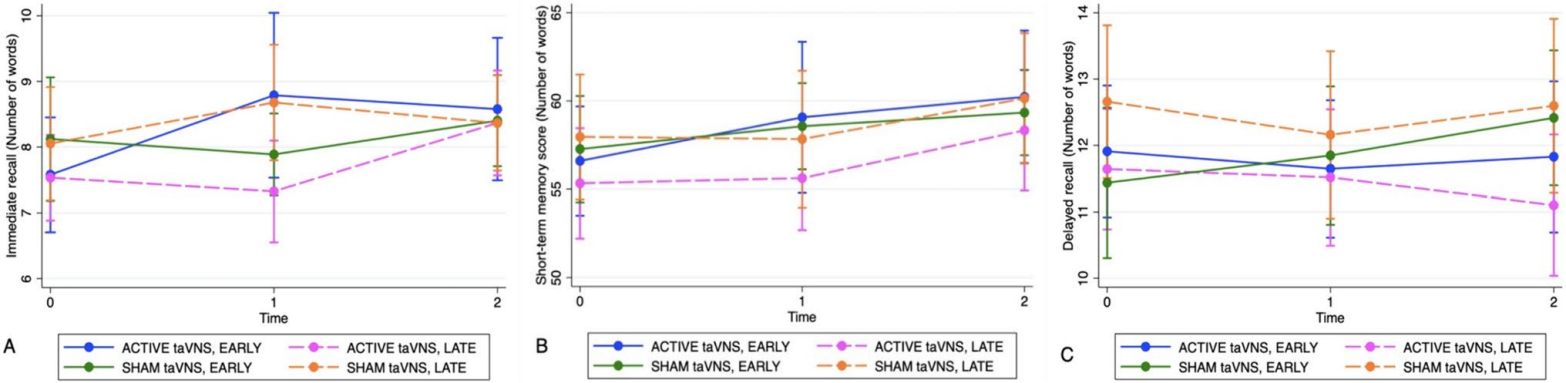
Predicted change in immediate recall (**A**), short-term memory score (**B**), and delayed recall (**C**) with 95% confidence intervals in the four groups over the course of the study. Estimates for each timepoint for each group were predictions from a mixed model including immediate memory or short-term memory score or delayed recall, time, intervention phase, age, sex, and the following interaction terms: time × intervention; time × phase; intervention × phase; time × intervention × phase

Table 4 summarizes the results of the analysis of the prespecified contrasts. For the active taVNS group, there was a statistical difference in the estimated means of immediate recall and short-term memory score between time 0 and time 1 in participants who underwent stimulation early (Table 4 and blue line in Fig. 3A, B) (immediate recall: *C* = 1.21, 95% CI 0.25–2.17; short-term memory score: *C* = 2.47, 95% CI 0.61–4.34), and between time 1 and time 2 in participants who received stimulation late (Table 4 and dashed pink line in Fig. 3A, B) (immediate recall: *C* = 1.04, 95% CI 0.28–1.8; short-term memory score: *C* = 2.71, 95% CI 0.38–5.04). Furthermore, as shown in Table 4 and Fig. 3A, B (blue line), the improvement in immediate recall and short-term memory score achieved in the early taVNS group between time 0 and time 1 persisted over the subsequent follow-up period as reflected by no change between time 1 and time 2 (immediate recall: *C* = − 0.21, 95% CI − 1.23 to 0.81; short-term memory score: *C* = 1.16, 95% CI − 0.89 to 3.21). Similarly, Table 4 and Fig. 3A, B (dashed pink line) show that in the late active taVNS groups, immediate recall and short-term memory score did not change between time 0 and time 1 during the first 2 weeks of waiting (immediate recall:* C* = − 0.21, 95% CI − 1.14 to 0.72; short-term memory score: *C* = 0.29, 95% CI − 2.42 to 3.00). For the sham taVNS groups, we observed no change in immediate recall or short-term memory score between time 0 and time 1 in participants who underwent sham taVNS early (Table 4 and green line in Fig. 3A, B) (immediate recall: *C* = − 0.24, 95% CI − 1.19 to 0.71; short-term memory score: *C* = 1.29, 95% CI − 1.56 to 4.15) and between time 1 and time 2 in participants who received sham taVNS late (Table 4 and dashed orange line in Fig. 3A, B) (immediate recall: *C* = − 0.31, 95% CI − 0.98 to 0.36; short-term memory score: *C* = 2.31, 95% CI   − 0.15 to 4.78).

**Table 4:** Analysis of prespecified contrasts with 95% confidence intervals between the four groups over the course of the study (*N* = 76) in immediate recall, short-term memory score, and delayed recall

Phase	Time group × phase	Immediate recall				Short-term memory score					Delayed recall				
		Contrast	Std. error	95% CI	*p* value	Contrast		Std. error	95% CI	*p* value	Contrast		Std. error	95% CI	*p* value
Early	1 vs 0 active taVNS	1.21	0.49	0.25–2.17	.013*	2.47		0.95	0.61–4.34	.009*	− 0.26		0.56	− 1.36 to 0.83	0.638
	1 vs 0 sham taVNS	− 0.24	0.48	− 1.19 to 0.72	.628	1.29		1.45	− 1.56 to 4.15	.374	0.41		0.34	− 0.25 to 1.07	0.220
	2 vs 1 active taVNS	− 0.21	0.52	− 1.22 to 0.81	.686	1.16		1.05	− 0.89 to 3.21	.267	0.18		0.29	− 0.39 to 0.75	0.530
	2 vs 1 sham taVNS	0.52	0.47	− 0.40 to 1.44	.273	0.77		1.08	− 1.34 to 2.89	.473	0.57		0.36	− 0.13 to 1.27	0.113
Late	1 vs 0 active taVNS	− 0.21	0.47	− 1.14 to 0.72	.661	0.29		1.38	− 2.42 to 3.00	.833	− 0.13		0.62	− 1.35 to 1.10	0.841
	1 vs 0 sham taVNS	0.63	0.45	− 0.26 to 1.51	.167	− 0.13		1.42	− 2.91 to 2.66	.930	− 0.5		0.46	− 1.41 to 0.41	0.280
	2 vs 1 active taVNS	1.04	0.39	0.28–1.80	.007*	2.71		1.19	0.38–5.04	.023*	− 0.42		0.46	− 1.32 to 0.48	0.364
	2 vs 1 sham taVNS	− 0.31	0.34	− 0.98 to 0.36	.360	2.31		1.26	− 0.15 to 4.78	.066	0.44		0.36	− 0.26 to 1.13	0.219

Estimates for each timepoint for each group were predictions from a mixed model including immediate recall/short-term memory score/delayed recall, time, intervention phase, age, sex, and the following interaction terms: time × intervention; time × phase; intervention × phase; time × intervention × phase

*Significant value (*p* < 0.05)

For delayed recall there was no change between relevant timepoints in any of the four taVNS groups (*p* > 0.113) (Table 4 and Fig. 3C).

### Main analysis: superiority of active against sham (placebo) taVNS

In the main analysis controlled for age and sex, for immediate recall, active taVNS was superior to sham taVNS in both early (*χ*^2^ = 4.41, *p* = 0.036) and late phases of stimulation (*χ*^2^ = 6.89, *p* = 0.009). For short-term memory score and delayed recall, active taVNS was not superior to sham in both early (short-term memory score: *χ*^2^ = 0.46, *p* = 0.497; delayed recall: *χ*^2^ = 1.07, *p* = 0.301) and late groups (short-term memory score: *χ*^2^ = 0.05, *p* = 0.819; delayed recall: *χ*^2^ = 2.16, *p* = 0.141).

**Fig. 4:**
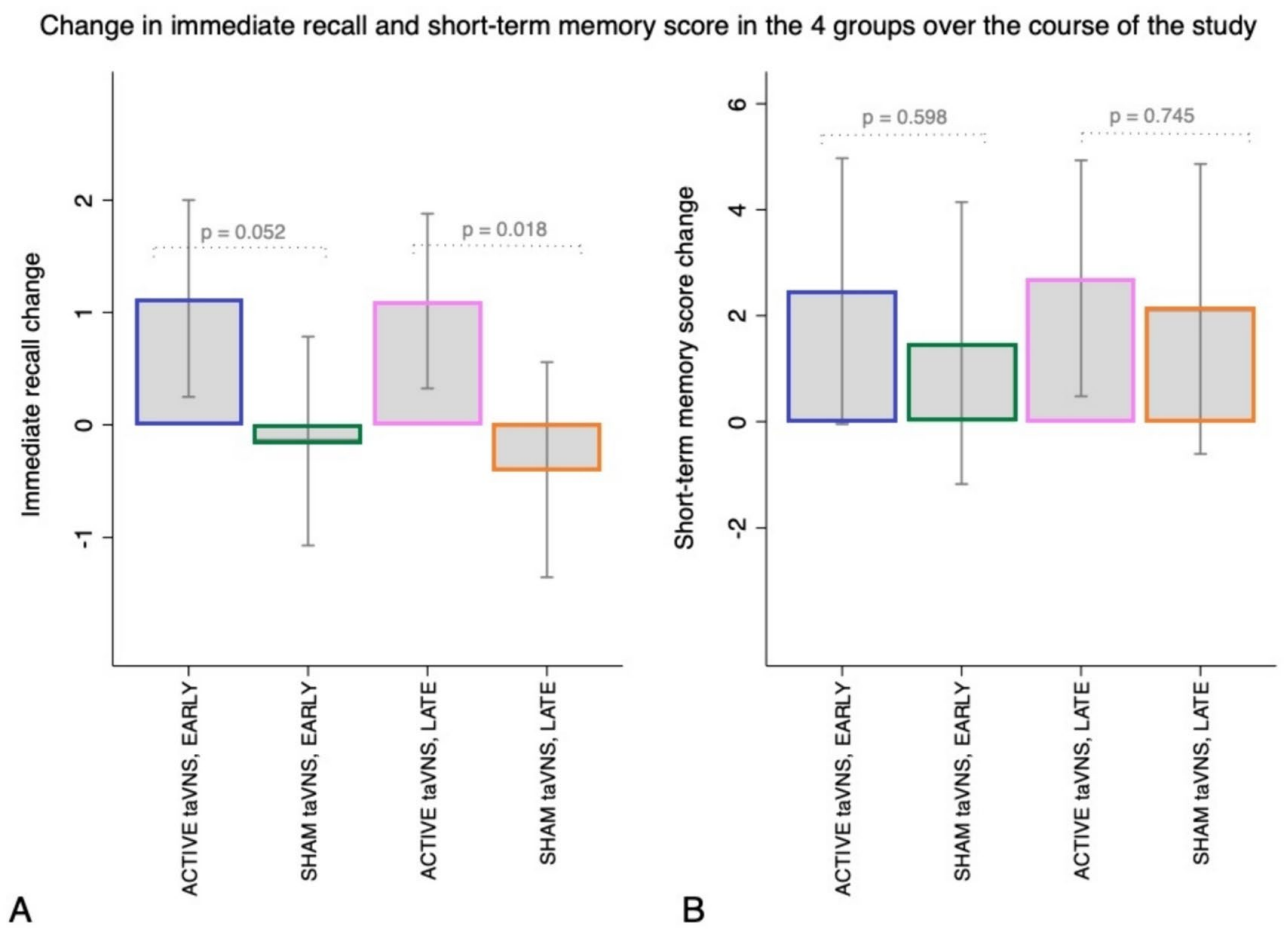
Differences between the pre- and post-intervention values of immediate recall (**A**) and short-term memory score (**B**) between time 1 and time 0 for early groups and between time 2 and time 1 for late groups

Figure 4 demonstrates the differences between the active and sham taVNS groups in either the early or late phase of calculated changes between pre- and post-intervention sessions. Figure 4A shows that, in the pairwise comparison, the change in immediate recall from pre- to post-intervention session is much greater in the active taVNS groups than in the sham taVNS groups, both in the early (*p* = 0.052) and late (*p* = 0.018) periods. Figure 4B demonstrates that, in the pairwise comparison, short-term memory score from pre- to post-intervention session did not differ between the early (*p* = 0.598) and late (*p* = 0.745) phases, although it was greater in the active taVNS groups than in the sham taVNS groups.

### Additional and sensitivity analyses

In additional models (Supplementary Tables SM1 to SM6), we controlled the analyses not only for age and sex but also for prescribed medication use and the presence of a self-reported diagnosed health issue. Immediate recall and short-term memory score results remained virtually unchanged. Statistical difference for immediate memory persisted, as did the superior effect of active taVNS. In further sensitivity analysis, after exclusion of five participants who reported taking antidepressants, anxiolytics, and hypnotics use, results remained consistent (Supplementary Tables SM7 and SM8).

### Harms and side effects

No serious side effects were reported. The most common undesired effect that participants reported was a light pain from the pressure of the clip at the site of stimulation (*N* = 26). Pain was rated on a scale from 1 to 10. The average pain score was 3.21 (SD 0.38, 95% CI 2.42–4.00) (see Table 5 for more detailed information).

**Table 5:** Reported pain in the four groups

Pain	Early		Late		*p* value
	Active taVNS	Sham taVNS	Active taVNS	Sham taVNS	
Yes	7	5	9	5	0.952
Pain scale	3 (1.67–4.33)	5.13 (− 0.32 to 10.57)	3.06 (2.17–3.95)	2.2 (0.58–3.82)	0.309

Data are presented as* n* or mean (95% CI)

## Discussion

In the present study, we investigated the impact of a 2-week course of taVNS on memory scores in 76 participants from a non-clinical population aged 18 to 75 years. We observed improvements in immediate recall and short-term memory score in participants who received active taVNS (in both early and late phases), whereas there was no improvement in participants who received sham taVNS (in both early and late phases). Additionally, the improvements achieved after the active taVNS in the early groups persisted over the subsequent follow-up period. Importantly, for immediate recall, the effect of active taVNS was superior to sham, but not so for short-term memory score. The results align with our hypothesis, suggesting that taVNS is likely to enhance memory, particularly immediate recall and short-term memory score. However, no improvement in delayed recall was observed.

To the best of our knowledge, this study is the first published research to investigate the effects of taVNS on memory in a well-controlled longitudinal design in non-clinical participants. As it was hypothesized that the biological pathways of the therapeutic effects of taVNS are similar to those of iVNS [56], we extend data from a clinical population to those from a non-clinical population. For example, Clark et al. [57] demonstrated improved verbal memory in patients with epilepsy after iVNS. Similarly, Ghacibeh et al. [58] found enhanced memory consolidation with iVNS. Chronic iVNS also showed positive effects on cognitive function in patients with Alzheimer’s disease [12, 13]. However, a 12-month follow-up study in patients with epilepsy reported no changes in objective memory scores after iVNS [59]. Helmstaedter et al. [60] observed a negative impact on memory processes, possibly due to high stimulation intensities (up to 3 mA). High stimulation intensities (up to 3 mA) may be effective in relieving epileptic seizures but have an inhibitory effect on memory consolidation [15].

Previous studies mainly focused on clinical populations, which can introduce bias due to underlying diseases affecting memory. Few short-term studies in healthy adults have explored the effects of taVNS on cognitive function, making comparisons with our 2-week study challenging. Nevertheless, some short-term studies showed positive effects of taVNS on tasks like divergent thinking [29], response selection [27], and emotion recognition from faces [61]. Jacobs et al. [25] reported that taVNS enhanced associative memory in the elderly even after a single session. Conversely, Mertens et al. [24] found no memory effects in young and middle-aged adults. It should be mentioned that in that study, stimulation was only delivered for 30 s during the consolidation phase of a memory task; however, 30 s of taVNS may be insufficient for a non-invasive device to effectively stimulate the vagal afferent pathway. Giraudier et al. [62] showed improved recollection-based memory after 23 min of taVNS. Kaan et al. [63] suggested taVNS may enhance item order memory.

**Fig. 5:**
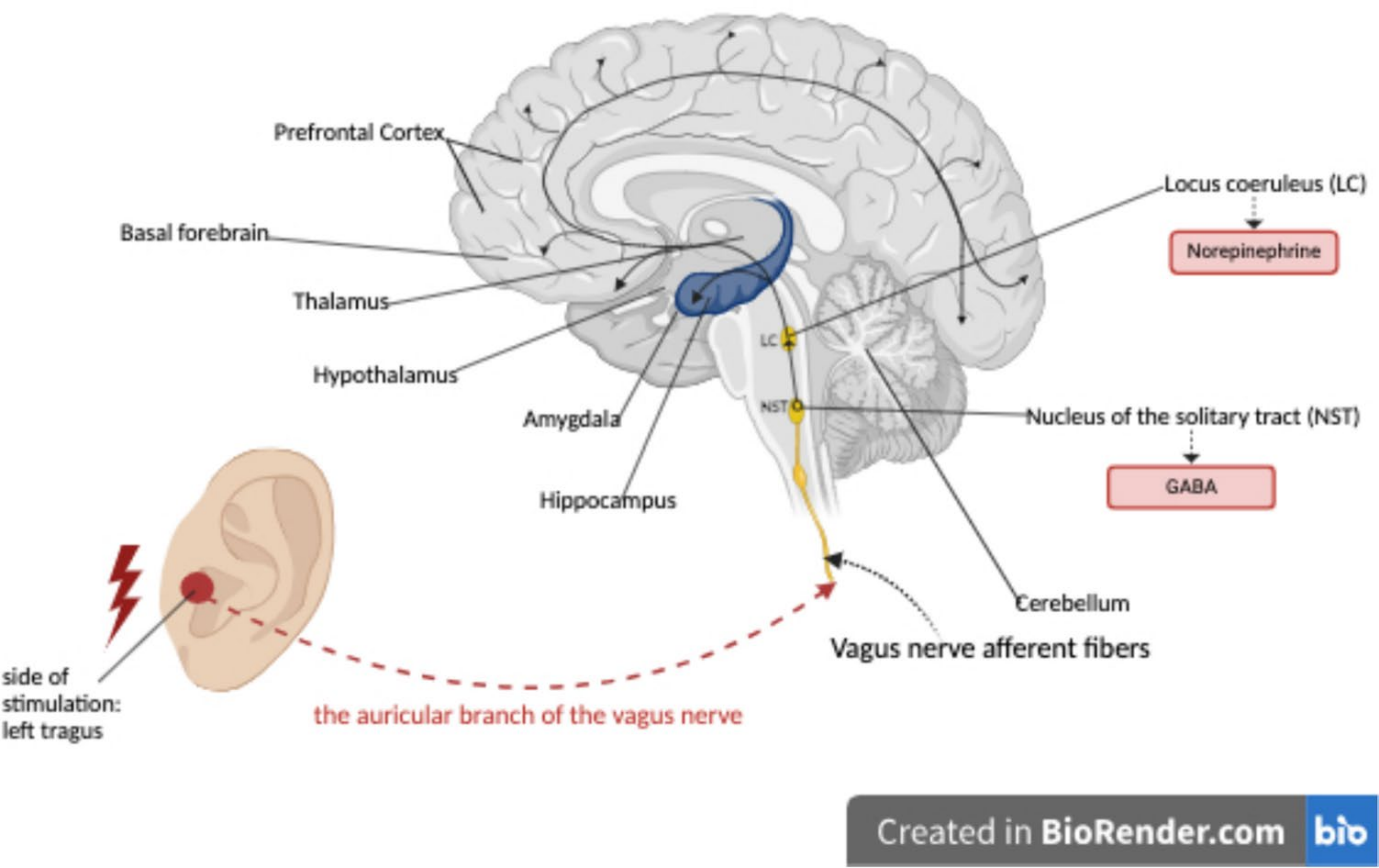
Schematic representation of the pathways involved in taVNS and their potential impact on brain areas associated with memory and other cognitive functions. Created in BioRender.com. The brain structures adapted from [9]

Several mechanisms and theories may explain our results. One possible mechanism suggests that the electrical impulses of taVNS stimulate afferent fibers, which primarily end in the NST. From the NST, projections extend to the LC, the brain’s primary noradrenergic center and main source of norepinephrine (NE) [6, 64]. The LC then sends widespread projections to various cortical and subcortical areas, including the amygdala and hippocampus [62, 65] (see Fig. 5 for an illustration of these pathways). This hypothesis is supported by a rat study showing increased NE levels in the hippocampus and cortex after VNS [66]. The LC-NE system is crucial for cognitive functions such as attention, executive functions, memory, and emotion recognition [64]. It is also possible that NE enhances memory by promoting long-term potentiation in the hippocampus, which is associated with increased synaptic plasticity, as demonstrated in the study by Zuo et al. [67]. Additionally, Colzato and Beste [68] conclude that tVNS increases gamma-aminobutyric acid (GABA), the primary inhibitory neurotransmitter [6], via activation of the NTS. It is important to note that, although evidence suggests that VNS increases levels of NE and GABA in the brain, most studies rely on indirect inferences rather than direct measurements of these neurotransmitters [64].

Furthermore, release of neurotransmitters like acetylcholine, serotonin, dopamine, and brain-derived neurotrophic factor (BDNF), as confirmed in neurophysiological studies [65], might contribute to taVNS’s mechanism of action. It is possible that taVNS reduces neuroinflammation through the cholinergic anti-inflammatory pathway [69], enhances blood flow to specific brain regions, influences peripheral hemodynamics [6], and modulates parasympathetic activity [70]. Additionally, our previous study [35] showed that 2 weeks of taVNS improved sleep scores, which could be linked to the activation of the ascending reticular activating system [71] known for its role in regulating the sleep–wake cycle. Since sleep is associated with improved memory performance, this could be an additional explanation for our results.

The absence of effects on delayed recall, despite observed effects on immediate and short-term memory, raises questions. This could be due to ceiling effects, because some participants already performed close to the maximum in delayed recall, and the mean scores across groups were generally high. Uttl [72] demonstrated that healthy adults can recall more than typically assessed by memory tests, suggesting susceptibility to ceiling effects, especially in the RAVLT. A lower baseline score in delayed recall may have allowed for more noticeable improvement. This emphasizes the need for memory tests that account for ceiling effects to accurately assess cognitive changes, particularly in delayed recall.

Important study limitations should be noted. Firstly, taVNS was self-administered, potentially affecting compliance. However, we believe, that the self-administered diary and daily questionnaires was a good choice to overcome this limitation. Secondly, the sensory innervation of the external ear is not only provided by the ABVN but also by the auriculotemporal nerve, a branch of the mandibular and therefore trigeminal nerve, and the greater auricular nerve [73]. According to the study by Peuker and Filler [38], the tragus (used in active taVNS) is only 45% innervated by the ABVN, whereas the cymba conchae has 100% of its fibers. Nevertheless, some fMRI studies claim that the tragus is the optimal site for stimulation [39]. Moreover, the tragus is also innervated by the great auricular nerve and the auriculotemporal nerve [38] so it is not necessarily solely the vagus nerve that is eliciting observed effects [74]. Thirdly, the use of the earlobe for sham stimulation, despite being considered anatomically free of vagal innervation [38], can be seen as a limitation. This choice is debated [75] because the earlobe has been used as part of a non-invasive transcutaneous brain stimulation technique known as cranial electrotherapy stimulation [76]. Therefore, it is not physiologically inert and can produce activation patterns similar to taVNS, potentially confounding results [47, 77]. Nonetheless, the earlobe remains the standard in auricular stimulation studies [46] because it should induce sensations similar to active condition without influencing the vagal afferents in the brainstem [68], thus controlling for most confounders such as unblinding [78]. Additionally, in our study, no statistically significant changes in memory performance were observed following sham taVNS. Fourthly, sample size was not determined on the basis of a formal a priori power analysis but in line with existing research. Fifthly, the participants may have been generally healthier than the general population, limiting generalizability. Lastly, not blinding the laboratory staff to the intervention group can be seen as a limitation, although this approach is commonly employed in studies. Future studies should aim to achieve double-blinding. Despite these limitations, our study featured a strong study design in a non-clinical population that enabled us to test both the within- and between-subject effects. Another strength is that we included a placebo and wait-list control group and ensured participants’ adherence through an anonymous box system. This approach maintained confidentiality and potentially honest reporting, enhancing the study’s accuracy.

## Conclusion

We showed that 2 weeks of active taVNS might have the potential to improve immediate recall and short-term memory score. Moreover, these effects have likely the potential to persist, as reflected by maintained improvement in immediate recall and short-term memory score over subsequent follow-ups. We observed no changes in delayed recall, which could be explained by the ceiling effect. In conclusion, the benefits and application of non-invasive vagus nerve stimulation, such as taVNS, go beyond treating clinical populations and could be scaled up to a population level to prevent the global burden of cognitive impairment and dementia. Our findings also highlight the importance of healthy vagal tone in the prevention of memory loss and mitigating cognitive ageing.

## Supplementary Information

Below is the link to the electronic supplementary material.

Supplementary file1 (DOCX 41 KB)

## Data Availability

Data can be made available upon request.
