# Correction to: Influence of a 2‑week transcutaneous auricular vagus nerve stimulation on memory: findings from a randomized placebo controlled trial in non‑clinical adults

**DOI:** 10.1007/s10286-024-01100-w

**Published:** 2025-01-15

**Authors:** Veronika Cibulcova, Julian Koenig, Marta Jackowska, Vera Kr Jandackova

**Affiliations:** 1 Department of Epidemiology and Public Health, Faculty of Medicine, University of Ostrava, Syllabova 19, Ostrava, 703 00 Czech Republic; 2 Department of Child and Adolescent Psychiatry, Psychosomatics and Psychotherapy, Faculty of Medicine, University of Cologne, University Hospital Cologne, Cologne, Germany; 3 Faculty of Psychology, SWPS University, Sopot, Poland; 4 Department of Human Movement Studies, Faculty of Education, University of Ostrava, Ostrava, Czech Republic

**Correction to: Clinical Autonomic Research (2024) 34:447–462** 10.1007/s10286-024-01053-0

The article “Influence of a 2‑week transcutaneous auricular vagus nerve stimulation on memory: findings from a randomized placebo controlled trial in non‑clinical adults”, written by Veronika Cibulcova, Julian Koenig, Marta Jackowska, and Vera Kr Jandackova., was originally published Online First without Open Access. After publication in volume 34, issue 4, page 447–462, the author decided to opt for Open Choice and to make the article an Open Access publication. Therefore, the copyright of the article has been changed to © The Author(s) 2024 and the article is forthwith distributed under the terms of the Creative Commons Attribution 4.0 International License, which permits use, sharing, adaptation, distribution and reproduction in any medium or format, as long as you give appropriate credit to the original author(s) and the source, provide a link to the Creative Commons licence, and indicate if changes were made. The images or other third party material in this article are included in the article’s Creative Commons licence, unless indicated otherwise in a credit line to the material. If material is not included in the article’s Creative Commons licence and your intended use is not permitted by statutory regulation or exceeds the permitted use, you will need to obtain permission directly from the copyright holder. To view a copy of this licence, visit http://creativecommons.org/licenses/by/4.0. Open access funding enabled and organized by Projekt DEAL.

