# Supplementary material for: Influence of a 2-week transcutaneous auricular vagus nerve stimulation on memory: findings from a randomized placebo controlled trial in non-clinical adults
Source: Clin Auton Res. 2024 Jul 22;34(4):447–62. doi: 10.1007/s10286-024-01053-0 (PMC11732881; doi:10.1007/s10286-024-01053-0)
Supplement: Supplementary file 1 — Supplementary file1 (DOCX 41 KB) [file 10286_2024_1053_MOESM1_ESM.docx]

**Additional analyses:**

**Supplementary Table 1** Analysis of contrasts with 95% confidence intervals in immediate recall between the 4 groups over the course of the study (N=76) controlled for use of prescribed medication (N=27). Estimates for each timepoint for each group were predictions from a mixed model including immediate recall, time, intervention phase, age, sex, prescribed medication and the following interaction terms: time × intervention; time × phase; intervention × phase; time × intervention × phase.

| **Immediate recall (controlled for use of precribed medication)** | | | | | |
| --- | --- | --- | --- | --- | --- |
| **Phase** | **Time I group × phase** | **Contrast** | **Std. error** | **95% CI** | ***p-value*** |
| EARLY | 1 vs 0 ACTIVE taVNS | 1.21 | 0.49 | 0.25 to 2.17 | ***.013*** |
|  | 1 vs 0 SHAM taVNS | -0.24 | 0.49 | -1.19 to 0.72 | *.628* |
|  | 2 vs 1 ACTIVE taVNS | -0.21 | 0.52 | -1.23 to 0.81 | *.685* |
|  | 2 vs 1 SHAM taVNS | 0.52 | 0.47 | -0.40 to 1.44 | *.268* |
| LATE | 1 vs 0 ACTIVE taVNS | -0.21 | 0.47 | -1.14 to 0.72 | *.661* |
|  | 1 vs 0 SHAM taVNS | 0.63 | 0.45 | -0.26 to 1.51 | *.167* |
|  | 2 vs 1 ACTIVE taVNS | 1.04 | 0.39 | 0.28 to 1.80 | ***.007*** |
|  | 2 vs 1 SHAM taVNS | -0.31 | 0.34 | -0.98 to 0.36 | *.360* |

**Supplementary Table 2** Analysis of contrasts with 95% confidence intervals in short-term memory score between the 4 groups over the course of the study (N=76) controlled for use of prescribed medication (N=27). Estimates for each timepoint for each group were predictions from a mixed model including short-term memory score, time, intervention phase, age, sex, prescribed medication and the following interaction terms: time × intervention; time × phase; intervention × phase; time × intervention × phase.

| **Short-term memory score (controlled for use of precribed medication)** | | | | | |
| --- | --- | --- | --- | --- | --- |
| **Phase** | **Time I group × phase** | **Contrast** | **Std. Error** | **95% CI** | ***p-value*** |
| EARLY | 1 vs 0 ACTIVE taVNS | 2.47 | 0.95 | 0.61 to 4.34 | ***.009*** |
|  | 1 vs 0 SHAM taVNS | 1.29 | 1.45 | -1.56 to 4.15 | *.374* |
|  | 2 vs 1 ACTIVE taVNS | 1.16 | 1.05 | -0.89 to 3.21 | *.267* |
|  | 2 vs 1 SHAM taVNS | 0.77 | 1.08 | -1.34 to 2.89 | *.475* |
| LATE | 1 vs 0 ACTIVE taVNS | 0.29 | 1.38 | -2.42 to 3.00 | *.833* |
|  | 1 vs 0 SHAM taVNS | -0.13 | 1.42 | -2.91 to 2.66 | *.930* |
|  | 2 vs 1 ACTIVE taVNS | 2.71 | 1.19 | 0.38 to 5.04 | ***.023*** |
|  | 2 vs 1 SHAM taVNS | 2.31 | 1.26 | -0.15 to 4.78 | *.066* |

**Supplementary Table 3** Analysis of contrasts with 95% confidence intervals in immediate recall between the 4 groups over the course of the study (N=75) controlled for use of precribed medication and presence of self-reported diagnosed health issue (N=43). Estimates for each timepoint for each group were predictions from a mixed model including immediate recall, time, intervention phase, age, sex, prescribed medication, self-reported diagnosed health issue and the following interaction terms: time × intervention; time × phase; intervention × phase; time × intervention × phase.

| **Immediate recall (controlled for use of precribed medication and presence of self-reported diagnosed health issue)** | | | | | |
| --- | --- | --- | --- | --- | --- |
| **Phase** | **Time I group × phase** | **Contrast** | **Std. Error** | **95% CI** | ***p-value*** |
| EARLY | 1 vs 0 ACTIVE taVNS | 1.28 | 0.51 | 0.28 to 2.28 | ***.013*** |
|  | 1 vs 0 SHAM taVNS | -0.24 | 0.48 | -1.19 to 0.72 | *.628* |
|  | 2 vs 1 ACTIVE taVNS | -0.22 | 0.55 | -1.30 to 0.86 | *.685* |
|  | 2 vs 1 SHAM taVNS | 0.54 | 0.47 | -0.39 to 1.46 | *.257* |
| LATE | 1 vs 0 ACTIVE taVNS | -0.21 | 0.47 | -1.14 to 0.72 | *.661* |
|  | 1 vs 0 SHAM taVNS | 0.63 | 0.45 | -0.26 to 1.51 | *.167* |
|  | 2 vs 1 ACTIVE taVNS | 1.04 | 0.39 | 0.28 to 1.80 | ***.007*** |
|  | 2 vs 1 SHAM taVNS | -0.31 | 0.34 | -0.98 to 0.36 | *.360* |

**Supplementary Table 4** Analysis of contrasts with 95% confidence intervals in short-term memory score between the 4 groups over the course of the study (N=75) controlled for use of precribed medication and presence of self-reported diagnosed health issue (N=43). Estimates for each timepoint for each group were predictions from a mixed model including short-term memory score, time, intervention phase, age, sex, prescribed medication, self-reported diagnosed health issue and the following interaction terms: time × intervention; time × phase; intervention × phase; time × intervention × phase.

| **Short-term memory score (controlled for use of precribed medication and presence of self-reported diagnosed health issue)** | | | | | | |
| --- | --- | --- | --- | --- | --- | --- |
| **Phase** | **Time I group × phase** | **Contrast** | **Std. error** | **95% CI** | ***p-value*** |  |
| EARLY | 1 vs 0 ACTIVE taVNS | 2.72 | 0.97 | 0.82 to 4.63 | ***.005*** |  |
|  | 1 vs 0 SHAM taVNS | 1.29 | 1.45 | -1.56 to 4.15 | *.374* |  |
|  | 2 vs 1 ACTIVE taVNS | 0.82 | 1.05 | -1.24 to 2.89 | *.435* |  |
|  | 2 vs 1 SHAM taVNS | 0.79 | 1.09 | -1.34 to 2.91 | *.468* |  |
| LATE | 1 vs 0 ACTIVE taVNS | 0.29 | 1.38 | -2.42 to 3.00 | *.833* |  |
|  | 1 vs 0 SHAM taVNS | -0.13 | 1.42 | -2.91 to 2.66 | *.930* |  |
|  | 2 vs 1 ACTIVE taVNS | 2.71 | 1.19 | 0.38 to 5.04 | ***.023*** |  |
|  | 2 vs 1 SHAM taVNS | 2.31 | 1.26 | -0.15 to 4.78 | *.066* |  |

**Supplementary Table 5** Analysis of contrasts with 95% confidence intervals in immediate recall between the 4 groups over the course of the study (N=76) controlled for use of hypnotics, anxiolytics and antidepressants (N=5). Estimates for each timepoint for each group were predictions from a mixed model including immediate recall, time, intervention phase, age, sex, prescribed hypnotics or anxiolytics or antidepressants and the following interaction terms: time × intervention; time × phase; intervention × phase; time × intervention × phase.

| **Immediate recall (controlled for use of hypnotics, anxiolytics and antidepressants)** | | | | | |
| --- | --- | --- | --- | --- | --- |
| **Phase** | **Time I group × phase** | **Contrast** | **Std. error** | **95% CI** | ***p-value*** |
| EARLY | 1 vs 0 ACTIVE taVNS | 1.21 | 0.49 | 0.25 to 2.17 | ***.013*** |
|  | 1 vs 0 SHAM taVNS | -0.24 | 0.48 | -1.19 to 0.72 | *.628* |
|  | 2 vs 1 ACTIVE taVNS | -0.21 | 0.52 | -1.23 to 0.81 | *.689* |
|  | 2 vs 1 SHAM taVNS | 0.51 | 0.47 | -0.41 to 1.43 | *.275* |
| LATE | 1 vs 0 ACTIVE taVNS | -0.21 | 0.47 | -1.14 to 0.72 | *.661* |
|  | 1 vs 0 SHAM taVNS | 0.63 | 0.45 | -0.26 to 1.51 | *.167* |
|  | 2 vs 1 ACTIVE taVNS | 1.04 | 0.39 | 0.28 to 1.80 | ***.007*** |
|  | 2 vs 1 SHAM taVNS | -0.31 | 0.34 | -0.98 to 0.36 | *.360* |

**Supplementary Table 6** Analysis of contrasts with 95% confidence intervals in short-term memory score between the 4 groups over the course of the study (N=76) controlled for use of hypnotics, anxiolytics and antidepressants (N=5). Estimates for each timepoint for each group were predictions from a mixed model including short-term memory score, time, intervention phase, age, sex, prescribed hypnotics or anxiolytics or antidepressants and the following interaction terms: time × intervention; time × phase; intervention × phase; time × intervention × phase.

| **Short-term memory score (controlled for use of hypnotics, anxiolytics and antidepressants)** | | | | | |
| --- | --- | --- | --- | --- | --- |
| **Phase** | **Time I group × phase** | **Contrast** | **Std. error** | **95% CI** | ***p-value*** |
| EARLY | 1 vs 0 ACTIVE taVNS | 2.47 | 0.95 | 0.61 to 4.34 | ***.009*** |
|  | 1 vs 0 SHAM taVNS | 1.29 | 1.45 | -1.56 to 4.15 | *.374* |
|  | 2 vs 1 ACTIVE taVNS | 1.16 | 1.05 | -0.89 to 3.21 | *.267* |
|  | 2 vs 1 SHAM taVNS | 0.77 | 1.08 | -1.34 to 2.87 | *.477* |
| LATE | 1 vs 0 ACTIVE taVNS | 0.29 | 1.38 | -2.42 to 3.00 | *.833* |
|  | 1 vs 0 SHAM taVNS | -0.13 | 1.42 | -2.91 to 2.66 | *.930* |
|  | 2 vs 1 ACTIVE taVNS | 2.71 | 1.19 | 0.36 to 5.04 | ***.023*** |
|  | 2 vs 1 SHAM taVNS | 2.31 | 1.26 | -0.15 to 4.78 | *.066* |

**Sensitivity analyses:**

**Supplementary Table 7** Analysis of contrasts with 95% confidence intervals in immediate recall between the 4 groups over the course of the study with exclusion of 5 participants who use hypnotics, anxiolytics and antidepressants (N=71). Estimates for each timepoint for each group were predictions from a mixed model including immediate recall, time, intervention phase, age, sex, and the following interaction terms: time × intervention; time × phase; intervention × phase; time × intervention × phase.

| **Immediate recall (exclusion of 5 participants who use hypnotics, anxiolytics and antidepressants)** | | | | | |
| --- | --- | --- | --- | --- | --- |
| **Phase** | **Time I group × phase** | **Contrast** | **Std. error** | **95% CI** | ***p-value*** |
| EARLY | 1 vs 0 ACTIVE taVNS | 1.21 | 0.49 | 0.25 to 2.17 | ***.013*** |
|  | 1 vs 0 SHAM taVNS | -0.25 | 0.52 | -1.26 to 0.76 | *.628* |
|  | 2 vs 1 ACTIVE taVNS | -0.21 | 0.52 | -1.23 to 0.81 | *.689* |
|  | 2 vs 1 SHAM taVNS | 0.48 | 0.50 | -0.50 to 1.46 | *.339* |
| LATE | 1 vs 0 ACTIVE taVNS | -0.52 | 0.50 | -1.51 to 0.46 | *.296* |
|  | 1 vs 0 SHAM taVNS | 0.53 | 0.47 | -0.39 to 1.46 | *.260* |
|  | 2 vs 1 ACTIVE taVNS | 1.14 | 0.43 | 0.30 to 1.99 | ***.008*** |
|  | 2 vs 1 SHAM taVNS | -0.13 | 0.31 | -0.75 to 0.48 | *.670* |

**Supplementary Table 8** Analysis of contrasts with 95% confidence intervals in short-term memory score between the 4 groups over the course of the study with exclusion of 5 participants who use hypnotics, anxiolytics and antidepressants (N=71). Estimates for each timepoint for each group were predictions from a mixed model including short-term memory score, time, intervention phase, age, sex, and the following interaction terms: time × intervention; time × phase; intervention × phase; time × intervention × phase.

| **Short-term memory score (exclusion of 5 participants who use hypnotics, anxiolytics and antidepressants)** | | | | | |
| --- | --- | --- | --- | --- | --- |
| **Phase** | **Time I group × phase** | **Contrast** | **Std. error** | **95% CI** | ***p-value*** |
| EARLY | 1 vs 0 ACTIVE taVNS | 2.47 | 0.95 | 0.61 to 4.34 | ***.009*** |
|  | 1 vs 0 SHAM taVNS | 1.44 | 1.54 | -1.58 to 4.45 | *.350* |
|  | 2 vs 1 ACTIVE taVNS | 1.16 | 1.05 | -0.89 to 3.22 | *.266* |
|  | 2 vs 1 SHAM taVNS | 0.47 | 1.11 | -1.70 to 2.64 | *.669* |
| LATE | 1 vs 0 ACTIVE taVNS | -0.52 | 1.47 | -3.40 to 2.36 | *.722* |
|  | 1 vs 0 SHAM taVNS | -0.40 | 1.49 | -3.32 to 2.52 | *.788* |
|  | 2 vs 1 ACTIVE taVNS | 2.57 | 1.34 | -0.06 to 5.20 | *.055* |
|  | 2 vs 1 SHAM taVNS | 2.60 | 1.31 | 0.04 to 5.16 | *.047* |

**Information:**

Influence of a 2-week transcutaneous auricular vagus nerve stimulation on memory: findings from a randomized placebo controlled trial in non-clinical adults

Clinical Autonomic Research

Veronika Cibulcova, Julian Koenig, Marta Jackowska, Vera Kr Jandackova

Correspondence: Veronika.Cibulcova@osu.cz, Syllabova 19, 703 00, Czech Republic
